# Uncovering Latent Tuberculosis Masquerading as Bartonella henselae Infection in a 12-Year-Old Patient

**DOI:** 10.7759/cureus.92017

**Published:** 2025-09-10

**Authors:** Tarannum Dhillon, Nicholas Pereira

**Affiliations:** 1 School of Medicine, Saint James School of Medicine, Arnos Vale, VCT; 2 Pediatrics, South Texas Health System Children’s, Edinburg, USA

**Keywords:** bartonella henselae, cat scratch disease, concurrent infections, latent infection, mycobacterium tuberculosis, pediatric, pediatric infection

## Abstract

Bartonella henselae, the causative agent of cat scratch disease (CSD), and Mycobacterium tuberculosis are two distinct pathogens that may affect the same patient, particularly in areas where both are common and risk factors overlap. We report the case of a 12-year-old Hispanic boy from South Texas, living near the USA-Mexico border, who presented with cervical swelling and mild periorbital edema for more than two weeks. Outpatient evaluation identified recent kitten exposure and positive Bartonella henselae IgM titers, leading to a diagnosis of CSD. Clindamycin was prescribed due to a documented penicillin allergy, although it is not considered first-line therapy. The patient’s lymphadenopathy persisted after completing treatment, prompting admission to our hospital for further evaluation. During hospitalization, azithromycin, the CDC-recommended first-line antibiotic, was started for CSD, and latent tuberculosis infection was also identified. Our case illustrates how an acute illness can dominate the clinical presentation, overshadowing an underlying latent condition, creating a diagnostic blind spot, thus reinforcing the need for comprehensive history-taking, systematic evaluation, and a broad differential diagnosis, especially in patients with multiple risk factors.

## Introduction

Cat scratch disease (CSD) is a zoonotic disease primarily associated with cats and the cat flea, Ctenocephalides felis, which remains the primary vector, despite emerging data suggesting potential spread through other arthropods [1]. Among the nearly 20 Bartonella species, Bartonella henselae is the most common cause of infection in humans [1]. The classic presentation is regional lymphadenopathy and fever, accompanied by swelling of the conjunctiva, eyelid, or surrounding skin, which occurs in up to 8% of cases [1]. Most infections are associated with kittens and stray cats, accounting for 92.4% of reported cases [2]. In the United States, about 13,000 cases are diagnosed annually, with children under 14 years representing 32.5% of cases and an incidence of 9.4 per 100,000 population [2]. Tuberculosis (TB) is caused by the Mycobacterium tuberculosis and is transmitted through the inhalation of respiratory droplets [3]. It remains a major global health concern. As of 2022, the WHO reported 10.6 million active cases worldwide, including 1.3 million in children [3]. Drug-resistant strains have made eradication difficult, as about 25% of the world's population is infected, and up to 10% may develop active disease [3]. Clinical features range from asymptomatic infection to severe illness. Symptoms are often nonspecific and include cough, fever, weight loss, night sweats, and malaise, with intrathoracic lymphadenopathy being more common in children and immunocompromised patients [3]. Although the United States has a low incidence (four cases per 100,000), most pediatric TB cases occur in children born abroad or with family members from other countries [4].

## Case presentation

A 12-year-old Hispanic boy from South Texas, living near the USA-Mexico border, was initially evaluated at an outpatient clinic. He presented with progressive swelling on the left side of his neck extending under the jawline and mild periorbital swelling of the left eye. The swelling had persisted for over two weeks with a one-day history of low-grade fever of 99.5°F; the patient denied constitutional and respiratory symptoms. He reported frequently playing and nuzzling with stray cats and kittens in his neighborhood, though he did not recall any recent scratches or bites. Initial laboratory evaluation showed elevated Bartonella henselae IgM titers. Based on these findings, CSD was diagnosed, and oral clindamycin was prescribed due to severe penicillin allergy. Although clindamycin is not first-line therapy for CSD, this decision was made outside our institution. Following completion of clindamycin therapy, his symptoms showed minimal improvement. As a result, he was referred to our hospital for further evaluation. On admission, he had left-sided cervical lymphadenopathy measuring approximately 2.5 cm, which was firm and mildly tender, as well as mild periorbital swelling. There was no erythema, rash, airway compromise, or other systemic findings. A more detailed history during hospitalization revealed early childhood exposure to his paternal grandfather from Mexico, who had active TB when the patient was two years old. At that time, the patient had a positive PPD skin test and a normal chest X-ray, but did not receive prophylactic TB therapy. His history raised suspicion of TB infection.

**Figure 1:**
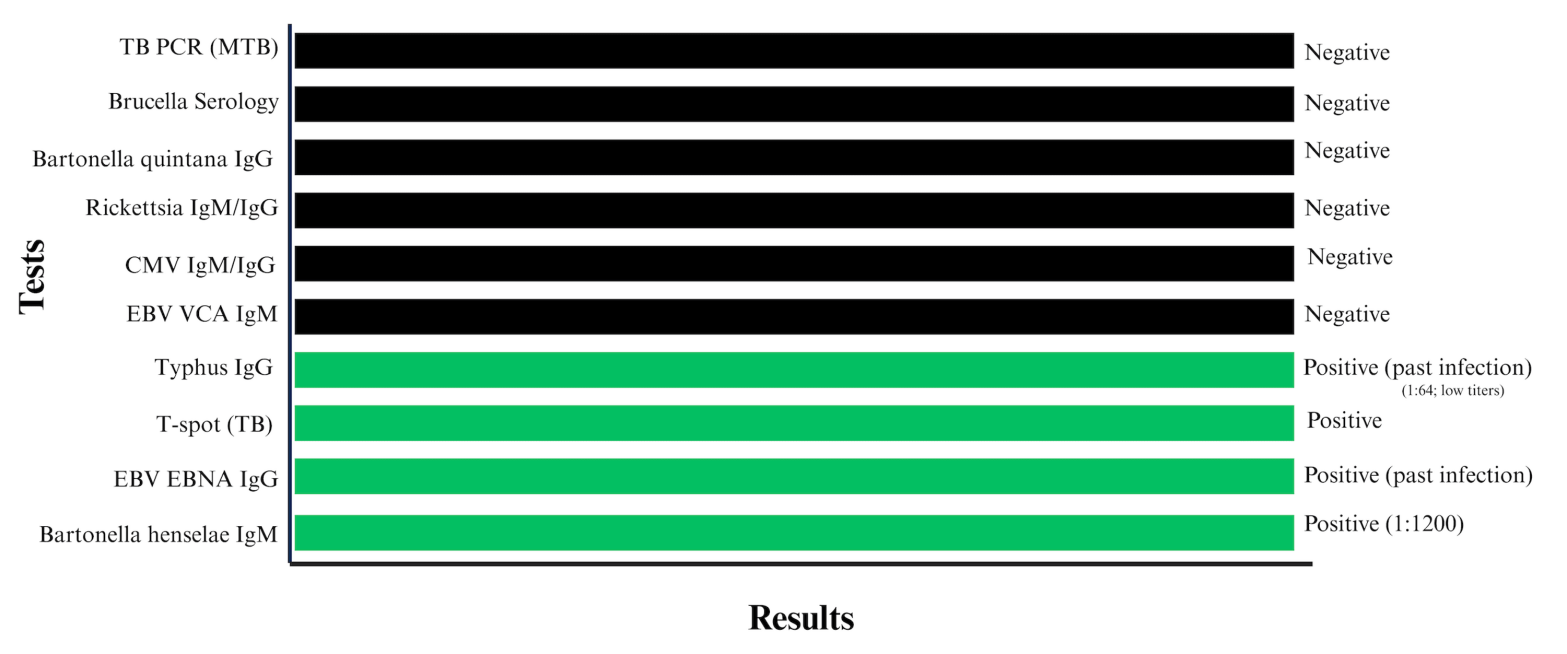
Summary of Serologic and Infectious Test Results EBV: Epstein–Barr virus; EBNA: Epstein–Barr nuclear antigen

At the hospital, serologic titers were repeated as part of a broader infectious workup. Bartonella henselae IgM remained positive, and inflammatory markers were elevated, with an ESR of 36 mm/hr (reference range: 3 to 15 mm/hr) and a procalcitonin level of 3.9 ng/mL (reference range: 0.10 - 0.49 ng/mL), supporting the diagnosis of CSD. EBV and Rickettsia IgG indicated past exposure, while CMV and Brucella titers were negative. Fine-needle aspiration (FNA) of the cervical lymph node was performed to exclude atypical mycobacterial infection or lymphoma. Cytology was negative for acid-fast bacilli and malignancy. Acid-fast bacilli (AFB) sputum smears and FNA aspirates were also negative for active TB. A T-Spot TB test (interferon-gamma release assay) was positive, confirming exposure to TB. The results of serologic and infectious testing are summarized in Figure 1.

**Figure 2:**
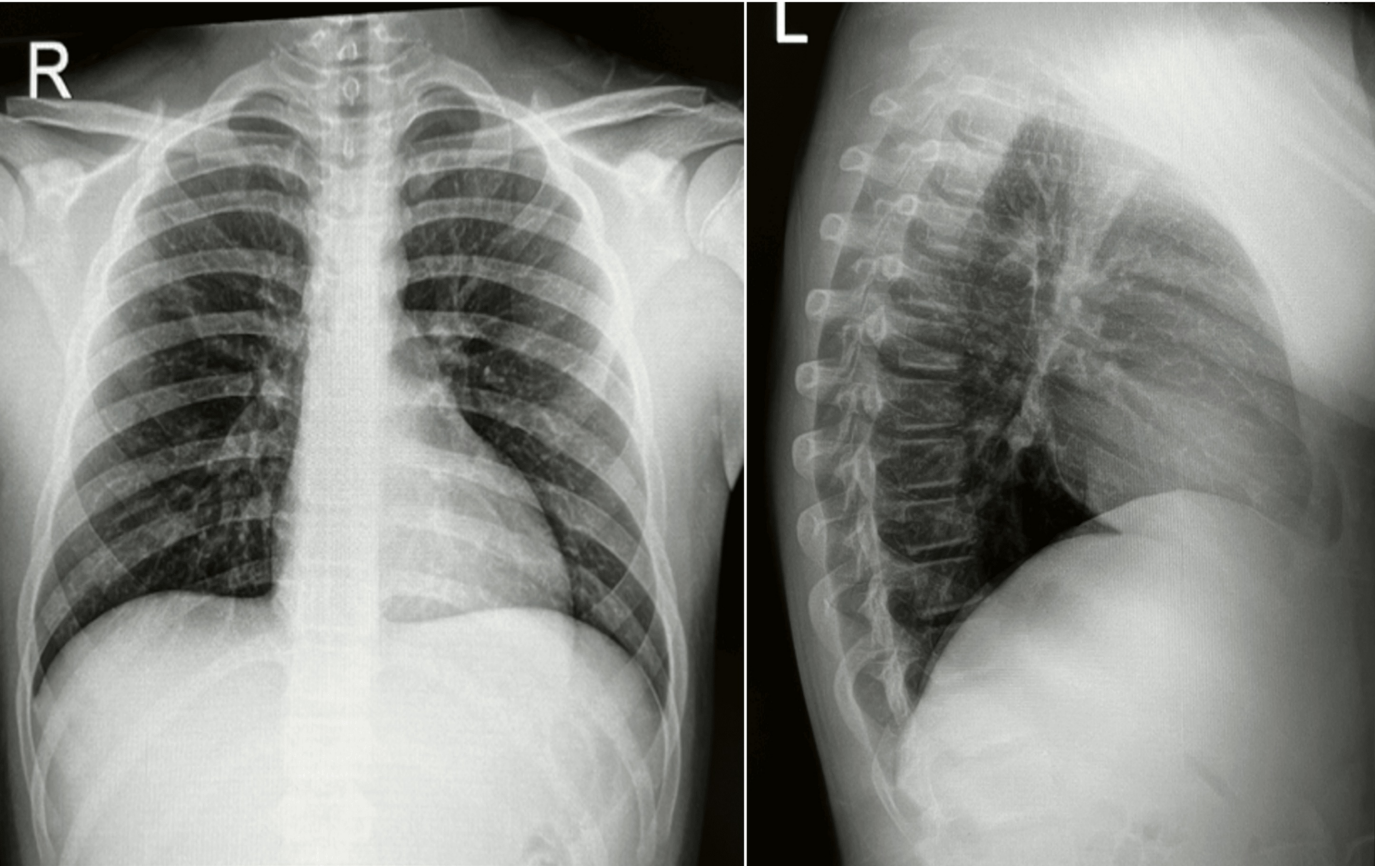
Normal Chest X-ray (Anteroposterior and Lateral Views) Showing No Acute Pulmonary Findings

Imaging revealed a clear chest X-ray with normal heart and mediastinal size, and no signs of pulmonary disease or active TB (Figure 2). Sinus X-ray showed clear paranasal sinuses and mastoid air cells, with no evidence of sinus disease. Based on the serology, imaging, and absence of active symptoms or pulmonary findings, latent TB infection was diagnosed.

During his hospital stay, our patient was started on azithromycin for CSD. He received 500 mg on the first day, followed by 250 mg daily for four days, in line with updated CDC recommendations that prefer azithromycin over clindamycin [5]. He also began treatment for latent TB infection with isoniazid 300 mg and rifampin 600 mg, both given orally once a day, as recommended by CDC guidelines [6]. Pyridoxine (vitamin B6) 50 mg was provided to prevent isoniazid-induced neuropathy, and acetaminophen 650 mg was administered every six hours for symptom relief, as needed. The planned regimen included five days of azithromycin and three months of isoniazid with rifampin.

The patient remained stable during hospitalization. Over the course of three days, the size of his cervical lymph node gradually decreased, which is the expected outcome of CSD when treated with azithromycin [1,2]. His CBC and CMP were within normal limits. He was discharged in stable condition with instructions to complete the remaining azithromycin course and continue latent TB therapy. Follow-up care was coordinated with his outpatient provider, pediatric infectious disease specialist, and the local public health department to reduce transmission risk and ensure continuity of care. The family was also counseled on the importance of completing therapy, attending follow-up visits, and monitoring and reporting new symptoms such as fever, swelling, cough, or weight loss.

## Discussion

This case demonstrates the difficulty in diagnosis when the prominent presentation of an acute infection draws clinical attention and masks an unrelated, asymptomatic, yet clinically significant underlying condition. Bartonella henselae is a fastidious, gram-negative bacillus that evades the immune system by forming protective vacuoles within CD34+ progenitor cells [1]. Its lipid A, an endotoxin, allows invasion of erythrocytes and endothelial cells, resulting in localized lymphadenopathy in 85-90% of cases [2]. In most immunocompetent individuals, CSD is typically mild and self-limiting [2]. In our case, persistence of lymphadenopathy and eventual hospitalization were likely related to the use of clindamycin, an antibiotic with limited efficacy for CSD. This is a clinical reminder that persistent lymphadenopathy should prompt evaluation for additional possible diagnoses, especially in high-risk areas. Mycobacterium tuberculosis is an acid-fast bacillus that establishes latency by forming granulomas, which involve macrophages and T lymphocytes [3]. In children, this containment is less effective, which increases the risk of reactivation or dissemination [3]. Latent TB infection is defined as an immune response to Mycobacterium tuberculosis in the absence of clinical evidence of active disease [7]. Although latent TB is usually asymptomatic, detection is important for both clinical management and public health as recent studies suggest that latency is not a static state and may involve periods of subclinical bacterial activity, even in healthy individuals [3]. Our case demonstrates a diagnostic blind spot rather than overlapping pathophysiology. The acute features of CSD accounted for the initial presentation, leading to a focus on a single diagnosis and overlooking coexisting latent conditions. The absence of systemic TB symptoms delayed further investigation. The patient’s history of household TB exposure was only identified after a detailed history was obtained during hospitalization, which enabled targeted screening and diagnosis. Early exposure to a family member with active TB was a significant risk factor. This serves as an important teaching point: recognizing such exposures through context-driven history is essential for providing optimal care even when initial findings seem straightforward. Acute infections like CSD can overshadow underlying silent diseases, emphasizing the need for a broad differential diagnosis and systematic evaluation of risk factors and exposures at each stage of care. The patient’s outpatient management also sheds light on practical challenges in care. He was initially prescribed clindamycin for CSD due to a documented penicillin allergy, even though clindamycin and penicillin are not first-line therapies according to CDC guidelines. This choice may reflect variations in clinical practice related to allergies, prescribing habits, or local availability of medications. His antibiotic regimen was updated during hospitalization to follow current evidence-based recommendations. Antimicrobial therapy can shorten the course of CSD, with azithromycin as the preferred first-line treatment [1,2,5]. Other agents, including rifampin, ciprofloxacin, gentamicin, and trimethoprim-sulfamethoxazole, have also shown efficacy [1,2]. Rifampin is used both as an alternative or adjunctive agent in refractory CSD and as a mainstay in the treatment of latent TB. Our patient did not have refractory CSD and rifampin was started solely for latent TB, which resulted in an unintentional overlap in therapy. This therapeutic intersection reinforces the teaching point that thorough clinical assessment is a crucial component of patient care to ensure the coordination of treatment plans and patient safety. Moreover, the geographic and epidemiological context was important in this case. Our patient lives near the USA-Mexico border, an area with higher TB rates and increased risk of cross-border exposure. In 2023, 90.1% of TB cases in the USA occurred among minorities, with the highest rates among Hispanic or Latino individuals [8]. Recognizing these geographic risks is important when developing a differential diagnosis and considering contributing factors. Awareness of these patterns should elicit early consideration of latent infections and guide timely testing and treatment. Reports of concurrent CSD and TB infections are limited, especially in pediatric patients. Eleftheriotis and Skopelitis described a case of CSD and active TB co-infection in an HIV-positive adult [9]. In immunocompromised individuals, including those with HIV, lymphadenopathy due to both active TB and Bartonella infection may occur together, especially with cat exposure [9]. In our case, an immunocompetent individual presented with both active CSD and latent TB. Our case shows that concurrent infections should be considered in individuals with significant exposure risks, regardless of immune status.

## Conclusions

In this case, an initial diagnosis of CSD ultimately revealed an unexpected coexistence with latent TB infection. This shows how an acute illness can shape the clinical impression and create diagnostic blind spots, allowing chronic conditions to go undetected without comprehensive assessment. Physicians should maintain vigilance for TB, particularly in endemic regions or in patients with a history of cross-border movement and other risk factors, even when symptoms suggest a more common diagnosis. Early detection and management of latent TB are essential to prevent progression to active disease and reduce community transmission. This case showcases that acute and chronic diseases can coexist and that careful history-taking, risk assessment, and systematic evaluation are necessary to improve outcomes, especially in pediatric patients.
